# Comparative Evaluation of Conventional and Digital Workflow Impressions for Implant-Supported Restorations

**DOI:** 10.3390/dj14020120

**Published:** 2026-02-18

**Authors:** Cristian Abad-Coronel, David Ruiz, Miguel Ángel Quelal, Diana Estrada, Nancy Mena Córdova, Paulina Aliaga

**Affiliations:** 1 Research Group in Digital Dentistry and CAD/CAM Materials, Faculty of Dentistry, Universidad de Cuenca, Cuenca 010107, Ecuador; 2 Prostodonthics Departament, Faculty of Dentistry, Universidad San Francisco de Quito, Quito 170901, Ecuador; drruizv@estud.usfq.edu.ec (D.R.);

**Keywords:** digital dentistry, CAD/CAM, implant-supported restoration, intraoral scanning, impression accuracy, marginal fit, passive adaptation

## Abstract

**Background:** Digital technologies, particularly CAD/CAM workflows, have transformed implant prosthodontics by improving the accuracy and efficiency of impression procedures, facilitating clinician–laboratory communication, and supporting the preservation of peri-implant tissues. **Objective:** To compare the three-dimensional accuracy (trueness) and passive fit of five conventional and digital impression techniques for fixed prostheses supported by two implants. **Methods:** An in vitro experimental study was conducted using a partially edentulous maxillary model with two implants supporting a three-unit zirconia bridge. Five impression workflows were evaluated: conventional techniques (open-tray and closed-tray, splinted and non-splinted) and digital impressions using plastic and titanium scan bodies. Three-dimensional accuracy was assessed by digital superimposition analysis, and passive fit was evaluated by marginal gap measurements using digital microscopy and ImageJ (version 1.54r) software. Statistical analyses were performed using exploratory ANOVA with Welch’s correction and Games–Howell post hoc tests (*p* < 0.05), complemented by effect size analysis. **Results:** Three-dimensional superimposition analysis revealed that digital impression workflows and the splinted conventional open-tray technique exhibited the highest trueness, with minimal spatial deviations relative to the reference model, together with the lowest marginal gap values (<1 µm). The non-splinted open-tray technique presented higher discrepancies (7.37 ± 0.94 µm), although all techniques remained within clinically acceptable tolerance ranges (60–150 µm). **Conclusions:** Under controlled in vitro conditions, both digital impression techniques and conventional splinted protocols achieve high three-dimensional accuracy and clinically acceptable passive fit for multi-implant-supported fixed prostheses. Digital workflows represent a predictable and efficient alternative, while conventional splinted impressions remain a reliable option depending on clinical and technological considerations.

## 1. Introduction

Over the past two decades, dentistry has undergone remarkable progress with the incorporation of digital technologies, particularly CAD/CAM systems [1]. These tools have transformed impression-taking and the fabrication of implant-supported restorations, enabling greater clinical precision and efficiency [2,3]. In addition, they improve peri-implant tissue protection and optimize communication between the clinician and the laboratory, representing a predictable alternative to traditional techniques [4]. Intraoral scanners (IOSs) capture high-resolution three-dimensional images that accurately replicate data previously obtained through conventional methods [5,6].

There are two main types of retention systems for implant-supported restorations, namely screw-retained and cement-retained, as well as hybrid combinations used in crowns, bridges, or complete prostheses [7,8]. The success of implant rehabilitation depends on achieving a passive fit that minimizes stress on prosthetic components and peri-implant tissues, preventing complications such as fractures, peri-implantitis, bone resorption, or even implant failure [9,10].

The conventional implant impression technique uses elastomeric materials and customized trays—either open or closed—to transfer the position of implants to the master model [11,12]. In multiple-unit rehabilitations, it is recommended to splint the copings with acrylic resin to reduce movement and improve the final fit [13,14,15]. This workflow remains the most widely used due to its low cost and accessibility [16]. For many clinicians, these techniques are familiar and reliable, especially in complex clinical cases [17]. Technological advances have promoted the use of intraoral scanners for digital impressions in implant-supported rehabilitations, aiming for greater accuracy, speed, predictability, and patient comfort [6,18,19]. Digital scanning eliminates errors associated with gypsum pouring and allows files to be stored indefinitely in STL, DXD, or OBJ formats [20]. Evaluating the quality of digital scans is essential to identify potential errors that may arise during the process, related to both the IOS hardware and software [21].

The accuracy of digital scanning depends on several factors, including scanner type, lighting conditions, operator experience, and implant characteristics [22,23]. Despite its advantages, the learning curve and high initial costs still limit its widespread adoption [24]. However, digital impressions enhance communication with the laboratory and enable the design, fabrication, and customization of restorations [25,26]. The choice between digital or analog impressions depends on multiple biomechanical, esthetic, anatomical, and economic factors [27,28,29]. It has been established that absolute passive fit is not always achievable. While some authors have suggested that a maximum misfit of up to 150 µm may be clinically acceptable [6], others have proposed a more restrictive threshold of approximately 60 µm [2]. These ranges have been widely cited in the literature as reference values for clinically acceptable passive fit [16].

Despite the advantages of digital impressions, comparative evidence remains limited, particularly in rehabilitations involving multiple splinted implants. This lack of consensus justifies the need for studies evaluating the accuracy and trueness between both techniques [12]. One of the critical aspects in implant-supported rehabilitation is the misfit and lack of passivity of the restoration; therefore, impressions must be accurate and precise to minimize clinical and laboratory risks [30].

To ensure the success and longevity of rehabilitations, achieving a passive fit that prevents stress on components and peri-implant tissues is essential, as it helps avoid complications such as fractures, loss of retention, bone resorption, peri-implantitis, and even implant loss [27].

The accuracy of the final implant model directly depends on the trueness and precision of the impression techniques and materials used [31,32]. Currently, analog impressions using transfers, customized trays, and addition silicones remain the most common [33]. However, digital impression systems with intraoral scanners (IOSs) offer high accuracy and immediate communication with the laboratory [34,35]. The precision depends on the scanning strategy employed to achieve a clinically acceptable fit, although trueness is not yet fully established and requires further investigation [24]. In implant-supported rehabilitation, digital scanning is crucial for accurately capturing implant positions and facilitating computer-aided design and manufacturing (CAD/CAM) of the final restoration [36].

Digital impressions offer significant clinical advantages: reduced risk of distortion, faster procedures, and improved patient experience by eliminating unpleasant tastes, gag reflexes, and potential allergic reactions. They also eliminate the need for trays, impression materials, and disinfection processes, simplifying the transfer of files to the laboratory [37]. However, scientific literature is still limited regarding direct comparisons between digital and analog techniques in multiple splinted implant rehabilitations [38]. To the best of our knowledge, limited preliminary studies have evaluated this impression protocol using next-generation intraoral scanners; therefore, the present investigation was designed as a pilot study to obtain initial comparative data under controlled conditions.

The primary objective of this in vitro study was to evaluate the three-dimensional accuracy (trueness) of conventional and digital impression techniques for multiple implant-supported fixed prostheses using a standardized superimposition analysis.

The secondary objective was to assess the passive fit of the resulting three-unit zirconia restorations by measuring marginal adaptation at the implant–abutment interface using digital microscopy.

The null hypothesis was that no significant differences would be observed between impression techniques in terms of three-dimensional accuracy or marginal fit.

## 2. Materials and Methods

### 2.1. Study Design

**Figure 1:**
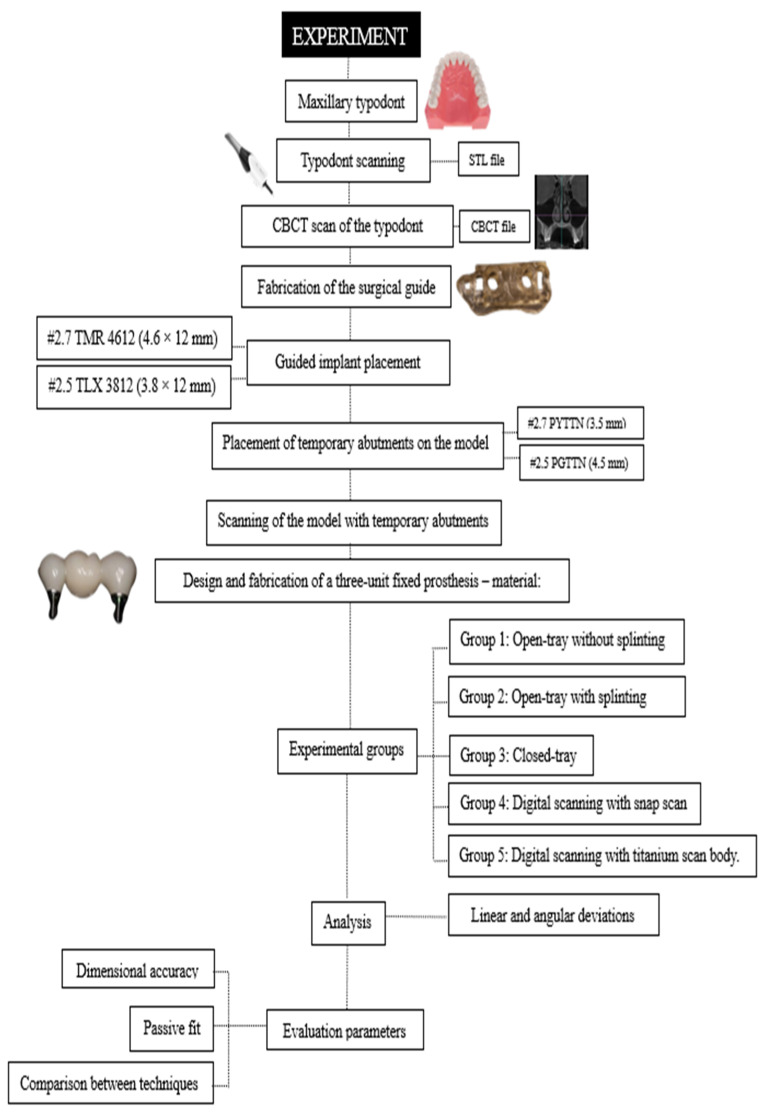
Flowchart of the experimental procedure. The diagram illustrates the sequential steps of the study, including master model design, CBCT acquisition, guided implant placement, temporary abutment positioning, conventional and digital impression workflows, prosthesis fabrication, and subsequent measurement protocols.

This experimental in vitro study was conducted using a standardized master model to compare different conventional and digital impression techniques for fixed prostheses supported by multiple implants. A three-unit fixed bridge supported by two implants was designed and evaluated in a partially edentulous maxillary model. The study aimed to assess the dimensional accuracy of the impression techniques through three-dimensional analysis, as well as the resulting passive fit of the fabricated prostheses as a clinically relevant outcome. The overall workflow of the experimental protocol is illustrated in Figure 1.

### 2.2. Materials

A partially edentulous maxillary master model made of ivorine material (typodont) was used. Two implants were placed using a guided surgical protocol, a BioHorizons Tapered Internal Implant TMR 4612 (4.6 × 12 mm; LOT 2204404), in position #2.7 and a BioHorizons Tapered Internal Implant TLX3812 (3.8 × 12 mm; LOT 2401218) in position #2.5 (BioHorizons, Birmingham, AL, USA).

Temporary abutments were used (included one Internal 3.5 mm Titanium Temporary Abutment Non-hexed, PYTTN; LOT 2302483 and one internal 4.5 mm Titanium Temporary Abutment Non-hexed PGTTN; LOT 2206222, BioHorizons, Birmingham, AL, USA).

The definitive prosthesis was fabricated from a monolithic zirconia disk (3D DML-3D PRO, Hunan, China). CAD/CAM design was performed using a dental specific software (Exocad 3.2, Darmstadt, Germany). Digital impressions were obtained using a high-end label intraoral scanner (Primescan, Dentsply Sirona, Bensheim, Germany). The surgical guide was generated by merging DICOM data from cone-beam computed tomography (CBCT) (Galileos SL, Bensheim, Germany) with STL files of the master model. Prior to marginal gap evaluation, the zirconia bridge was seated on the corresponding abutments/analogs under standardized passive conditions. The restoration was positioned and gently seated using controlled manual pressure until complete seating was visually confirmed. No torque was applied, and no fit-checking materials were used, to avoid introducing additional variables and to allow direct assessment of the implant–abutment interface. This seating protocol was applied consistently for all experimental groups. The single monolithic zirconia three-unit bridge was used for all experimental models to eliminate prosthesis-related variability. The restoration was seated under standardized passive conditions, without torque, using gentle manual pressure.

**Figure 2:**
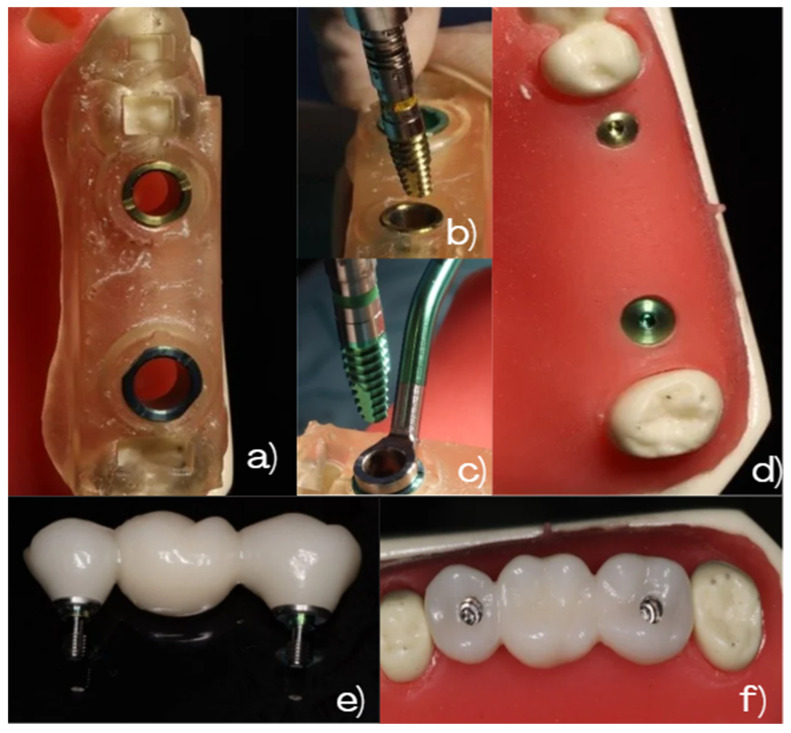
Surgical and prosthetic workflow on the master model. (**a**) Surgical guide with metallic sleeves used for guided implant placement. (**b**,**c**) Guided placement of implants in positions #2.5 and #2.7, respectively. (**d**) Final implant positions following guided placement. (**e**) Three-unit fixed zirconia bridge (#2.5–#2.6–#2.7). (**f**) Occlusal view of the fixed zirconia bridge seated on the ivorine master model.

The surgical protocol and the resulting prosthetic rehabilitation are illustrated in Figure 2, showing the guided implant placement, final implant positions, and the three-unit zirconia-fixed bridge on the ivorine model.

### 2.3. Experimental Groups

Five experimental groups (G1–G5) were established according to the impression technique and the type of abutment or scan body used (Table 1). A single standardized master model was employed, as is commonly adopted in in vitro accuracy studies, to eliminate anatomical variability and to ensure that any observed deviations were attributable exclusively to the impression workflow rather than to differences in implant position, angulation, or inter-implant distance.

As a pilot in vitro study, the primary objective was not to detect clinically meaningful differences in a confirmatory manner, but rather to explore the behavior of different conventional and digital impression workflows under controlled conditions and to estimate variability for the design of future confirmatory investigations. Accordingly, the number of repetitions per group (*n* = 5) was selected to support exploratory analysis and feasibility assessment, consistent with the objectives of a pilot study.

Each impression was considered an independent technical experimental unit within the controlled in vitro setting. Given the use of a single master model and repeated measurements obtained under identical conditions, the data do not represent independent biological replicates. Therefore, the statistical analyses were intended to be interpreted as descriptive and exploratory, with an emphasis on relative differences between workflows rather than on definitive inferential conclusions.

Due to the in vitro design and the absence of independent biological replication, a conventional a priori sample size calculation and statistical power analysis were not applicable. To provide additional context regarding the magnitude of the observed differences between workflows, effect sizes were calculated and reported alongside descriptive statistical analyses.

### 2.4. Procedures

#### 2.4.1. General Procedure

##### Impression Procedures

All impressions were performed on the same standardized master model to ensure identical anatomical conditions across all experimental groups and to eliminate confounding factors related to morphological variability. Five independent impressions were obtained for each technique (*n* = 5 per group; total = 25). Each impression and the resulting working model were considered the experimental unit for analysis.

#### 2.4.2. Conventional Impression Techniques (G1–G3)

For the conventional impression groups (G1–G3), impressions were made with the abutments positioned according to the manufacturer’s recommendations. Custom trays were fabricated in acrylic resin, and impressions were taken using addition silicone (Putty Base/Catalyst (Ivoclar Vivadent, Schaan, Liechtenstein).

In the splinted group (G2), the impression transfers were splinted using Pattern Resin LS acrylic resin (GC America Inc, Alsip, IL, USA), reinforced with chromium–nickel wire (Morelli, Sorocaba, SP, Brazil). After polymerization, the splint was sectioned and rejoined to minimize polymerization shrinkage.

**Figure 3:**
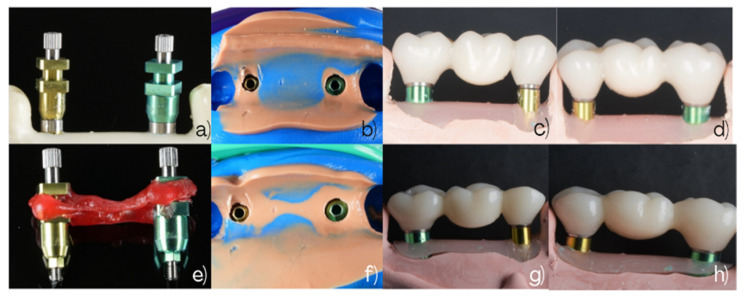
Conventional open-tray impression workflows. Group 1 (non-splinted direct transfers): (**a**) Buccal view of 3.5 mm and 4.5 mm direct pick-up transfers. (**b**) Occlusal view of the analog impression. (**c**) Buccal view of the three-unit fixed zirconia bridge (#2.5–#2.6–#2.7) connected to the corresponding analogs on the working model. (**d**) Palatal view of the same bridge on the working model. Group 2 (splinted direct pick-up transfers): (**e**) Buccal view of 3.5 mm and 4.5 mm direct pick-up transfers splinted prior to impression. (**f**) Occlusal view of the analog impression. (**g**) Buccal view of the three-unit fixed zirconia bridge connected to the corresponding analogs on the working model. (**h**) Palatal view of the same bridge on the working model.

**Figure 4:**
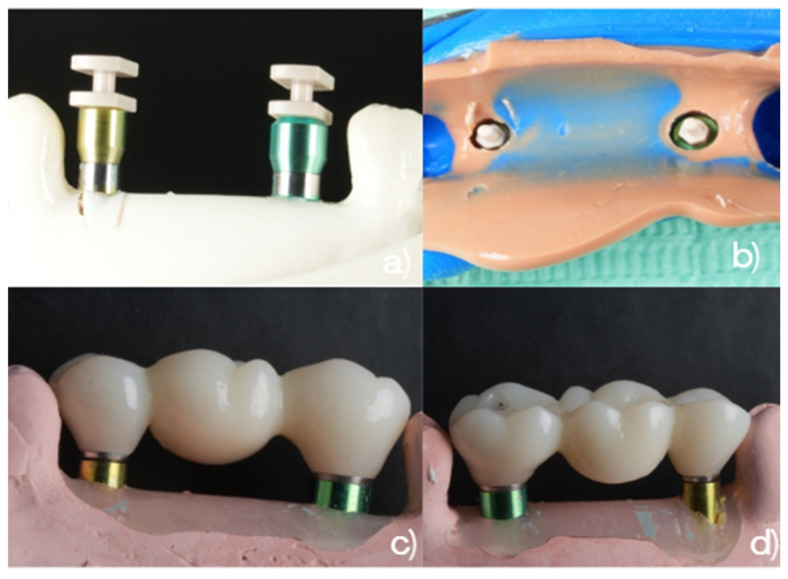
Conventional open-tray impression workflows. Group 3 (non-splinted direct pick-up copings): (**a**) Buccal view of 3.5 mm and 4.5 mm direct pick-up copings. (**b**) Occlusal view of the analog impression. (**c**) Buccal view of the three-unit fixed zirconia bridge (#2.5–#2.6–#2.7) connected to the corresponding analogs on the working model. (**d**) Palatal view of the same bridge on the working model.

The analog impression procedures and the resulting working models for Groups 1 and 2 are illustrated in Figure 3. The workflow for Group 3, using snap-copings, is illustrated in Figure 4.

#### 2.4.3. Digital Impression Techniques (G4–G5)

Digital impressions for Groups 4 and 5 were obtained using an intraoral scanner (Primescan, Dentsply Sirona, Bensheim, Germany), following the manufacturer’s recommended scanning protocol for multiple implant restorations. Depending on the group, either plastic or titanium scan bodies were used. The generated STL files were exported to a dental specific software (Exocad 3.2, Darmstadt, Germany) for digital model fabrication. Plastic and titanium scan bodies were selected for comparison because they represent the two most commonly available and clinically used scan body materials provided by implant manufacturer.

**Figure 5:**
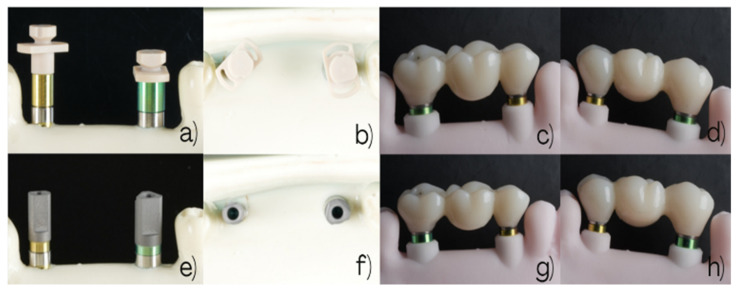
Digital impression workflows using scan bodies. Group 4 (plastic snap scan bodies): (**a**) Palatal view of the scan bodies positioned on the implants. (**b**) Occlusal view of 3.5 mm and 4.5 mm snap scan bodies. (**c**) Buccal view of the scan bodies. (**d**) Palatal view of the three-unit fixed zirconia bridge (#2.5–#2.6–#2.7) seated on the implants and connected to the corresponding analogs on the working model. Group 5 (titanium scan bodies): (**e**) Palatal view of the scan bodies positioned on the implants. (**f**) Occlusal view of 3.5 mm and 4.5 mm titanium scan bodies. (**g**) Buccal view of the scan bodies. (**h**) Palatal view of the three-unit fixed zirconia bridge seated on the implants and connected to the corresponding analogs on the working model.

The clinical and laboratory workflows for Groups 4 and 5 are illustrated in Figure 5.

### 2.5. Analysis Methods

#### 2.5.1. Three-Dimensional Superimposition and Trueness Analysis

To evaluate the three-dimensional accuracy of the different impression techniques, a digital superimposition analysis was performed using dedicated metrology software (OraCheck^®^, Cyfex, Zürich, Switzerland). A reference dataset was obtained by scanning the definitive three-unit zirconia prosthesis seated on the original master model, which served as the control condition.

For each experimental group, the same definitive prosthesis was subsequently seated on the corresponding working model derived from the respective impression technique and digitally scanned following a standardized acquisition protocol. The resulting datasets were individually superimposed onto the reference dataset using a best-fit alignment algorithm, allowing the assessment of spatial deviations attributable exclusively to the impression and model fabrication workflow.

Trueness and accuracy were quantified by calculating linear and angular deviations between the implant positions and prosthetic abutments in three-dimensional space. Trueness was defined as the degree of agreement between the experimental datasets and the reference model and was quantified using three-dimensional digital superimposition analysis. Precision was evaluated based on the consistency and variability of repeated measurements obtained within each experimental group.

#### 2.5.2. Microscopic Evaluation of Implant–Abutment Interfaces

In addition to the digital analysis, a microscopic evaluation was performed to assess the buccal and palatal aspects of the implant–abutment interfaces. Prior to marginal gap measurements, the digital microscope was calibrated using a certified reference scale, and analysis software (Image J software National Institutes of Health, Bethesda, MD, USA) was calibrated accordingly. Marginal gap values were obtained through calibrated digital measurements using the same software, ensuring traceability and measurement accuracy.

Marginal gap measurements were recorded at three predefined reference points (mesial, middle, and distal) on both the buccal and palatal aspects of each implant-supported restoration. All measurements were performed by a single calibrated examiner following a standardized protocol. To ensure measurement consistency, each reference point was measured repeatedly, and the mean value was used for statistical analysis, confirming high intra-examiner repeatability under the controlled in vitro conditions of the study.

**Figure 6:**
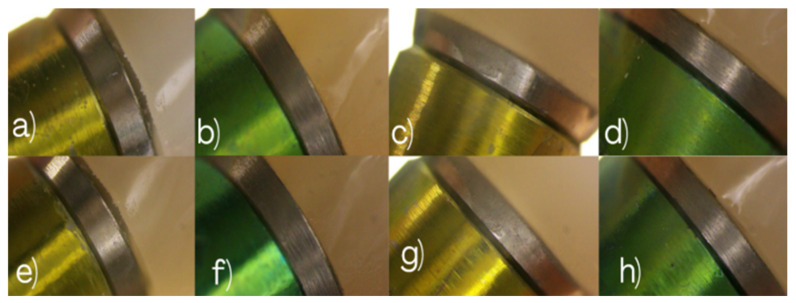
Digital microscopy images illustrating marginal gap assessment at the implant–abutment interface for conventional open-tray impression workflows. Group 1 (non-splinted direct pick-up transfers): (**a**) Buccal view of implant #2.5, (**b**) buccal view of implant #2.7, (**c**) palatal view of implant #2.5, and (**d**) palatal view of implant #2.7. Group 2 (splinted direct pick-up copings): (**e**) Buccal view of implant #2.5, (**f**) buccal view of implant #2.7, (**g**) palatal view of implant #2.5, and (**h**) palatal view of implant #2.7.

The figures illustrating marginal gap measurements are provided to document the measurement workflow, while numerical values reported in the Results section were derived directly from the software output. High-magnification images of implants #2.5 and #2.7 were obtained for Groups 1 and 2, providing qualitative and quantitative information on marginal adaptation and interface continuity. Representative microscopic images are presented in Figure 6.

#### 2.5.3. Evaluated Parameters

The following parameters were analyzed:

(1) Dimensional accuracy (trueness): Three-dimensional deviations between the reference dataset and the experimental datasets obtained from each impression technique.
(2) Passive fit of the prosthesis: Marginal adaptation and seating behavior of the three-unit fixed bridge on the master model, assessed through both digital and microscopic analyses.
(3) Comparison between techniques: Differences in accuracy and passive fit between conventional and digital impression workflows.

#### 2.5.4. Microscopic Evaluation of Marginal Fit

Marginal fit measurements were obtained using a digital microscope (AmScope Biological Research Microscope Irvine, CA, USA), 40×–2000× magnification, equipped with 5 megapixels camera. Image acquisition and measurement analysis were performed using an analysis software (ImageJ, National Institutes of Health, Bethesda, MD, USA).

**Figure 7:**
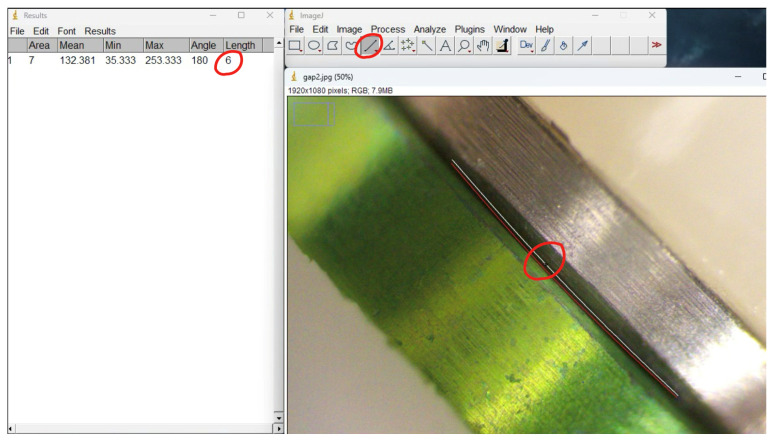
Screenshot of the marginal gap measurement procedure performed using analysis software (ImageJ Bethesda, MD, USA) following microscope calibration. The measurement line corresponds to the distance between the implant–abutment interface, and the numerical value is directly obtained from the software results window. This image illustrates the digital measurement workflow rather than a visual estimation of the gap.

Marginal discrepancies were measured in micrometers (µm) at three predefined reference points—mesial, middle, and distal—on both buccal and palatal aspects of the implant–abutment interface. For each specimen, the mean value of the recorded measurements was calculated and used for statistical analysis as seen in Figure 7.

**Figure 8:**
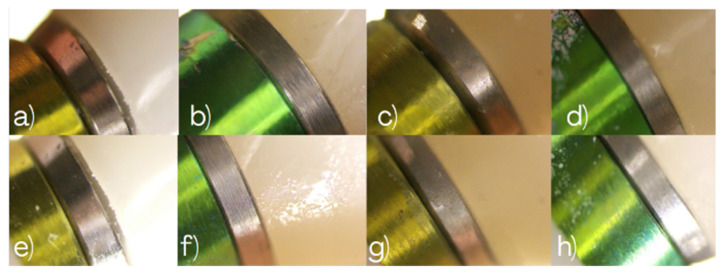
Digital microscopy images illustrating marginal gap assessment at the implant–abutment interface for the evaluated impression workflows. Group 3 (snap copings): (**a**) Buccal view of implant #2.5, (**b**) buccal view of implant #2.7, (**c**) palatal view of implant #2.5, and (**d**) palatal view of implant #2.7. Group 4 (digital impression with snap scan bodies): (**e**) Buccal view of implant #2.5, (**f**) buccal view of implant #2.7, (**g**) palatal view of implant #2.5, and (**h**) palatal view of implant #2.7.

**Figure 9:**
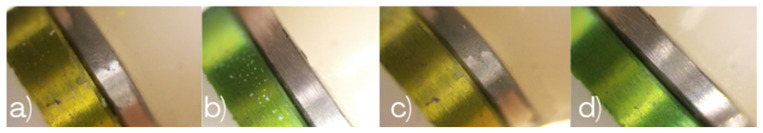
Digital microscopy images illustrating marginal gap assessment at the implant–abutment interface for the digital impression workflow using titanium scan bodies (Group 5). (**a**) Buccal view of implant #2.5, (**b**) buccal view of implant #2.7, (**c**) palatal view of implant #2.5, and (**d**) palatal view of implant #2.7.

This microscopic assessment allowed quantitative evaluation of marginal adaptation and verification of whether the observed discrepancies remained within the clinically acceptable tolerance ranges reported in the literature. Representative microscopy images illustrating the buccal and palatal aspects of implants #2.5 and #2.7 across all experimental groups are shown in Figure 6, Figure 8 and Figure 9, providing a comprehensive visual comparison of the implant–abutment interface and the seating behavior achieved with each impression technique.

### 2.6. Statistical Analysis

This section describes the statistical procedures applied for the analysis of marginal gap values; the results derived from these procedures are presented in Section 3. Data processing was performed using SPSS v27 software (IBM Corp., Armonk, NY, USA), and the level of significance was set at *p* < 0.05.

Marginal fit values were summarized using descriptive statistics, including mean, standard deviation, 95% confidence intervals, minimum, and maximum values. Data normality was assessed using the Shapiro–Wilk test, and variance homogeneity was evaluated with Levene’s test.

Due to the in vitro study design and the use of a single standardized master model, the multiple measurements obtained for each impression technique do not constitute independent biological observations. Therefore, conventional inferential statistical tests were applied in an exploratory and descriptive manner to identify patterns and relative differences between techniques under controlled conditions, rather than to support population-level inference.

Group comparisons were performed using one-way ANOVA with Welch’s correction, followed by Games–Howell’s post hoc tests. To complement these analyses and to better characterize the magnitude of the observed differences between impression techniques, effect sizes were calculated and reported.

## 3. Results

Group 1 exhibited the highest marginal gap values and the greatest variability (mean ± SD: 7.369 ± 0.949 µm). Group 3 showed intermediate discrepancies (2.915 ± 0.278 µm). In contrast, Groups 2, 4, and 5 demonstrated minimal marginal gaps and very limited dispersion (0.671 ± 0.021 µm, 0.891 ± 0.044 µm, and 0.659 ± 0.090 µm, respectively), indicating a more consistent and reproducible passive fit.

The measured marginal gap values ranged between <1 µm for the best-performing groups and values below 150 µm for all experimental groups, remaining within the clinically acceptable tolerance thresholds reported in the literature. Specifically, previous studies have indicated that marginal discrepancies of up to approximately 150 µm may be clinically acceptable [6], whereas more restrictive thresholds of around 60 µm have also been proposed [2,16]. ANOVA results revealed statistically significant differences in marginal fit among all groups (*p* < 0.05). The effect size analysis showed an extremely large effect, indicating that the impression technique accounted for more than 97% of the variance in marginal gap (η^2^ = 0.978, ω^2^ = 0.971).

**Figure 10:**
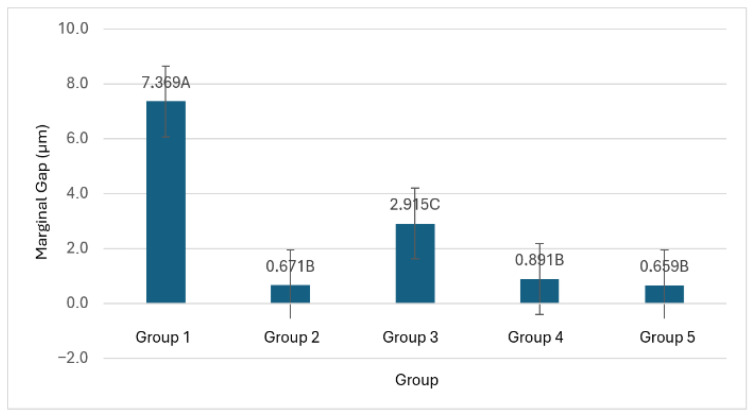
Bar chart illustrating the mean marginal gap values for each experimental group. Error bars represent the 95% confidence intervals. Identical letters indicate groups that did not differ significantly according to exploratory one-way ANOVA with Welch’s correction followed by the Games–Howell post hoc test.

The distribution of marginal gap values across the evaluated impression techniques has been illustrated. Group 1 presented higher mean marginal gap values compared with the other workflows. Groups 2, 4, and 5 showed lower mean discrepancies with overlapping confidence intervals, while Group 3 exhibited intermediate values. Differences between groups are presented descriptively, highlighting relative trends among workflows under the controlled in vitro conditions of this pilot study (Figure 10).

The multiple comparison test revealed homogeneous subsets corresponding to three performance levels. The first subset included Groups 2, 4, and 5, which showed no statistically significant differences (*p* ≥ 0.05). The second subset corresponded to Group 3, which exhibited intermediate values and statistically significant differences (*p* < 0.05) compared with the other groups. The third subset consisted of Group 1, which showed the highest discrepancies in marginal fit, with statistically significant differences compared to all other groups (*p* < 0.05).

**Figure 11:**
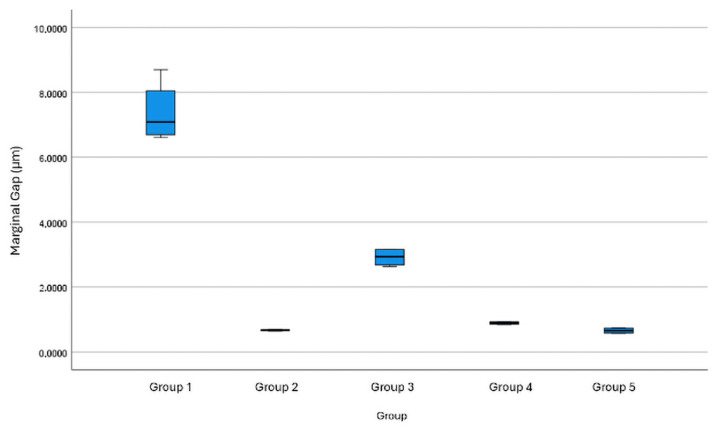
Box plot illustrating the distribution of marginal gap values (µm) for each experimental group. Boxes represent the interquartile range (IQR), the horizontal line within each box indicates the median value, and the whiskers denote the minimum and maximum observed values.

The distribution and variability of marginal gap measurements across the experimental groups have been illustrated. Group 1 presented a wider spread of values, while Group 3 showed an intermediate range of dispersion. In contrast, Groups 2, 4, and 5 exhibited marginal gap values concentrated within a narrower range, indicating lower variability among measurements (Figure 11).

**Figure 12:**
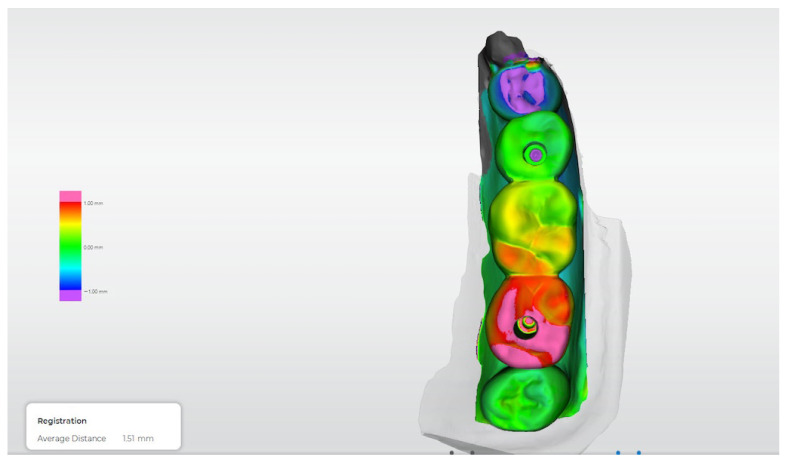
Three-dimensional digital superimposition analysis for Group 1 (conventional open-tray, non-splinted technique). Color-coded deviation map displays positive and negative spatial deviations relative to the reference dataset across the implant platforms and prosthetic surfaces.

**Figure 13:**
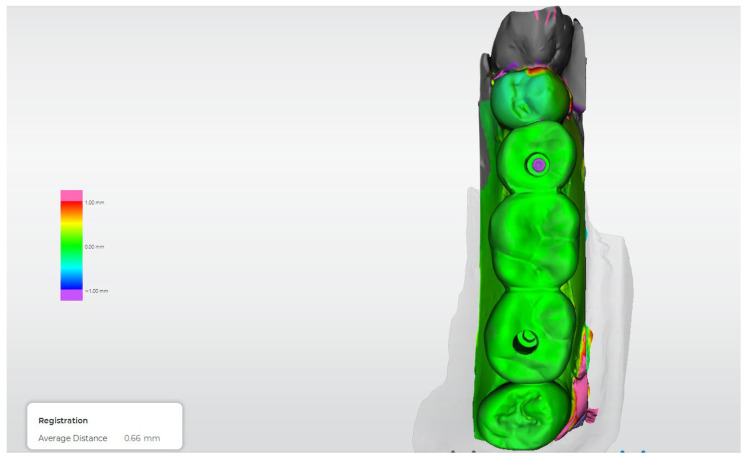
Three-dimensional digital superimposition analysis for Group 2 (conventional open-tray, splinted technique). Color-coded deviation map shows positive and negative spatial deviations relative to the reference dataset across the implant platforms and prosthetic surfaces.

**Figure 14:**
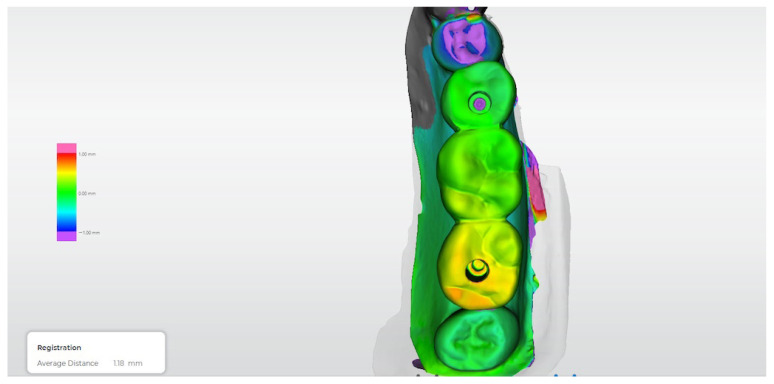
Three-dimensional digital superimposition analysis for Group 3 (conventional closed-tray technique with snap copings). Color-coded deviation map displays positive and negative spatial deviations relative to the reference dataset across the implant platforms and prosthetic surfaces.

**Figure 15:**
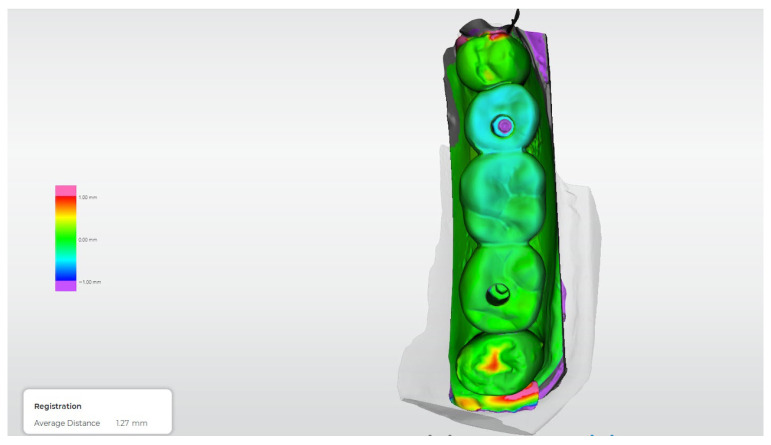
Three-dimensional digital superimposition analysis for Group 4 (digital impression using snap scan bodies). Color-coded deviation map displays positive and negative spatial deviations relative to the reference dataset across the implant platforms and prosthetic surfaces.

**Figure 16:**
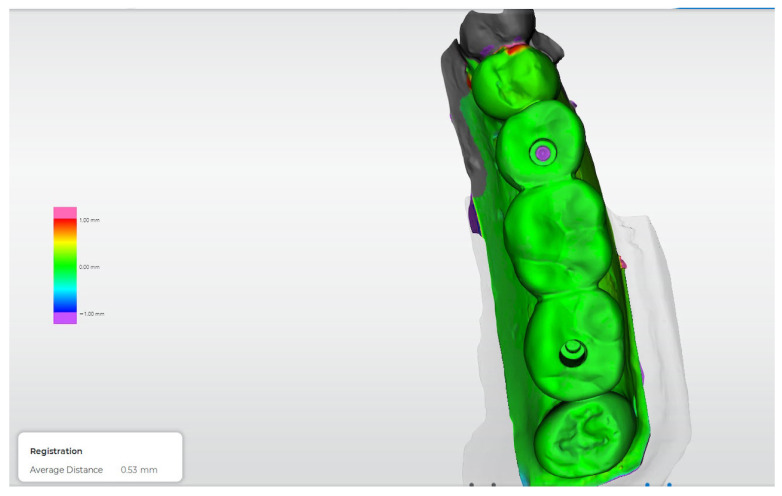
Three-dimensional digital superimposition analysis for Group 5 (digital impression using titanium scan bodies). Color-coded deviation map displays positive and negative spatial deviations relative to the reference dataset across the implant platforms and prosthetic surfaces.

Figure 12, Figure 13, Figure 14, Figure 15 and Figure 16 present representative three-dimensional superimposition maps illustrating the deviation patterns relative to the reference dataset for each experimental group and providing a qualitative comparison of the trueness associated with the different impression techniques.

## 4. Discussion

The present study aimed to compare conventional and digital impression techniques in terms of trueness, accuracy, and passive fit by evaluating different protocols, including open-tray and closed-tray conventional impressions, as well as intraoral scanning with various scan body designs. Within the present in vitro framework, trueness was assessed using three-dimensional digital superimposition analysis, whereas passive fit was evaluated as a functional expression of impression accuracy through marginal gap measurements at the implant–abutment interface. Regarding the study hypothesis, although statistically significant differences were detected among the evaluated impression techniques, the observed differences were quantitatively small and remained within clinically acceptable limits. Therefore, under the controlled in vitro conditions of this study, the null hypothesis of no clinically relevant differences in passive fit between the tested workflows was not rejected. The findings demonstrated that both the conventional open-tray splinted technique and the digital impression workflows achieved the most favorable marginal fit values (<1 µm). In contrast, the conventional open-tray non-splinted technique exhibited the greatest discrepancies, although all values remained within the commonly reported clinical tolerance range (60–150 µm). These results reinforce the concept that splinting and digital workflows enhance dimensional accuracy in implant-supported rehabilitations.

Beyond marginal fit assessment, the incorporation of three-dimensional superimposition analysis allowed a more comprehensive evaluation of trueness and spatial accuracy. The deviation maps revealed distinct patterns among the different impression techniques, which were consistent with the quantitative findings. Digital workflows and the splinted conventional technique exhibited more homogeneous deviation distributions, indicating improved positional fidelity relative to the reference dataset. Conversely, the non-splinted conventional technique showed greater localized deviations, particularly around the implant platforms, suggesting cumulative transfer inaccuracies. This qualitative–quantitative concordance supports the validity of the superimposition approach as a robust method for assessing impression accuracy.

From a clinical standpoint, these findings are relevant within the context of a controlled in vitro model, as precise transfer of implant positions is a prerequisite for achieving prosthetic passivity under idealized conditions. Additionally, although workflow efficiency was not directly evaluated in the present study, digital workflows have been reported in the literature to offer advantages in operational efficiency by reducing error-prone intermediate steps and facilitating clinician–laboratory communication. Nevertheless, the conventional open-tray splinted technique remains a well-established and reliable alternative, particularly in clinical settings with limited access to digital technologies or where minimizing initial investment is a priority.

As with all in vitro investigations, several limitations must be acknowledged when extrapolating these results to clinical practice. The absence of intraoral variables—such as saliva, soft tissue dynamics, patient movement, implant number and angulation, or access limitations—eliminates factors that may affect impression accuracy under real clinical conditions. Furthermore, the inclusion of only two parallel implants in a posterior segment restricts the applicability of these findings to more complex clinical scenarios, such as full-arch rehabilitations or cases with significant implant divergence, where error accumulation may be more pronounced.

In this context, the present results should be interpreted as controlled evidence supporting the reliability of digital impression protocols and conventional splinted techniques under standardized conditions. Future in vivo studies incorporating more complex implant configurations and longitudinal follow-up are required to further validate these findings.

Advances in digital technology have substantially transformed implant prosthodontics. The introduction of high-resolution intraoral scanners and CAD/CAM workflows has provided an efficient alternative to traditional open- or closed-tray impression techniques. Michelinakis et al. [3] reported that digital workflows eliminate critical error-prone steps, such as impression material handling, stone pouring, and transfer manipulation, thereby increasing data fidelity. In agreement with Rutkūnas et al. [6] and Papaspyridakos et al. [35], the digital groups in the present study demonstrated trends toward lower marginal discrepancies, supporting the reliability of intraoral scanning in partial implant-supported rehabilitations.

However, the absence of statistically significant differences in all comparisons suggests that digital techniques cannot yet be regarded as a universal replacement for conventional methods. Flügge et al. [13] and Albanchez–González et al. [16] emphasized that digital impression accuracy depends on multiple factors, including scanner type, scanning strategy, and arch extension. Under relatively simple configurations, such as two parallel implants supporting a three-unit bridge, digital systems appear to reach optimal performance. Accordingly, the statistical findings should be interpreted as controlled descriptive evidence highlighting relative differences between workflows, rather than as definitive inferential conclusions.

Conversely, conventional open-tray splinted impressions have long been considered the gold standard for multiple implant situations due to improved transfer stability. Gallucci et al. [11] demonstrated enhanced passive fit using splinted techniques compared with closed-tray approaches. The present findings are consistent with this evidence, showing smaller discrepancies for splinted impressions than for non-splinted ones, although in some cases values were slightly higher than those obtained through digital workflows. This reinforces the concept that conventional splinted impressions remain clinically reliable and relevant.

It is widely accepted that no technique guarantees a completely passive fit. Katsoulis et al. [10] and Albanchez–González et al. [16] reported that marginal discrepancies between 60 and 150 µm are clinically acceptable. Digital microscopy combined with software analysis allowed precise quantification of marginal gaps at mesial, distal, buccal, and palatal sites, confirming that all techniques tested remained within this range. The tendency of digital techniques to approach the lower threshold of discrepancy reflects their technical accuracy under standardized in vitro conditions, relative to non-splinted conventional impressions.

Figueras–Álvarez et al. [9] emphasized that even minor discrepancies can induce biomechanical stresses that may lead to long-term complications. The absence of drastic differences among techniques observed in this study suggests that clinical success depends not only on the choice between digital or analog workflows but also on correct protocol execution and operator experience. This observation aligns with Schmidt et al. [34], who underscored the influence of the human factor on both digital and conventional impression reproducibility.

Workflow efficiency has been identified in previous studies as an additional variable of interest in the comparison between digital and conventional impression workflows. Immediate file transmission to the laboratory reduces errors associated with transport, disinfection, and material deformation. Revilla–León et al. [36] highlighted that this immediacy improves clinician–technician communication and optimizes fabrication times. Although the present investigation was conducted in vitro, the consistency and predictability of the digital results support these reported advantages.

Despite the limitations, the present findings contribute meaningful evidence to the existing body of knowledge on implant impression techniques. The observed trend toward higher accuracy in digital workflows, particularly when using scan bodies and high-resolution scanners, aligns with reports by Ben–Izhack et al. [28] and Rutkūnas et al. [29]. From a clinical perspective, these results should be interpreted cautiously and in an integrative manner, recognizing that the objective is not to replace one technique with another, but rather to identify the clinical contexts in which each offers specific advantages. The measured discrepancies should be interpreted as relative indicators of seating accuracy within a standardized implant–abutment system under controlled in vitro conditions, rather than as absolute clinical marginal gaps. The submicron discrepancies reported in this study represent relative deviations measured at the implant–abutment interface under controlled in vitro conditions and should not be interpreted as clinical cement space or functional marginal gaps.

Conventional splinted impressions remain highly reliable, particularly in environments with limited digital infrastructure or cost constraints. In contrast, digital impressions represent a precise, efficient, and patient-friendly alternative, supporting their progressive adoption in routine clinical practice.

In summary, the results of this study, together with current literature, indicate that digital impression techniques—especially when combined with scan bodies and high-resolution scanners—provide high technical accuracy and favorable passive fit under controlled in vitro conditions for implant-supported splinted prostheses. Nonetheless, conventional splinted techniques continue to be supported by decades of clinical evidence and maintain full relevance in contemporary implant prosthodontics.

The true value of this investigation lies in demonstrating that clinical decision-making should not be framed as a dichotomy between digital and conventional approaches, but rather as a strategic integration of both, adapted to case-specific characteristics, patient conditions, and practitioner resources. Modern oral rehabilitation thus emerges as a hybrid scenario in which digital technologies advance rapidly while conventional methods continue to serve as a foundation of predictability and safety.

Limitations. A relevant limitation of this study is that each impression technique was evaluated using a single standardized master model, from which multiple measurements were obtained at different implant sites and perspectives. Although these repeated measurements enabled precise estimation of marginal discrepancies, they do not constitute independent biological replicates, and the limited number of impressions per group restricts statistical generalizability. Accordingly, the findings should be interpreted within the context of a controlled in vitro model and considered exploratory in nature.

All procedures and measurements were performed by a single calibrated operator to ensure methodological consistency and to minimize inter-operator variability; however, this may represent a potential source of operator-related bias. Examiner blinding was not implemented, although the use of standardized protocols and objective digital measurement tools was intended to mitigate this limitation. An additional limitation of this study is the use of a single zirconia bridge for repeated seating on multiple working models. Although seating was performed under passive, non-loaded conditions, minor alterations at the analog interface cannot be completely excluded.

Finally, while multiple measurements per specimen improved internal consistency, formal assessment of inter-operator or test–retest reliability was not performed and should be considered in future investigations.

## 5. Conclusions

Within the limitations of this pilot in vitro study, the results indicate that digital impression techniques achieve high three-dimensional accuracy (trueness) and clinically acceptable passive fit for implant-supported fixed prostheses under standardized laboratory conditions.

Conventional impression techniques, particularly open-tray protocols using splinted transfer abutments, also demonstrated favorable trueness and marginal adaptation outcomes, supporting their continued validity within controlled experimental settings.

Accordingly, the findings of this pilot investigation should be interpreted as preliminary and exploratory, highlighting relative differences between impression workflows rather than definitive clinical superiority. These results may assist in informing the design of future confirmatory in vivo studies and should not be extrapolated directly to long-term clinical decision-making.

## Figures and Tables

**Table 1:** Distribution of the experimental groups according to the impression technique and type of abutment.

Group	Impression Technique	Tray Type	Splinting	Abutment Type	Materials Used
G1	Conventional	Open plastic tray	No	3.5 mm Regular Direct Pick-Up Coping (PYRDC, LOT 2403954); 4.5 mm Regular Direct Pick-Up Coping (PGRDC, LOT 2404983); analogs: PYIA and PGIA	Putty Base/Catalyst (Ivoclar Vivadent); Type IV dental stone
G2	Conventional	Open plastic tray	Yes	3.5 mm Regular Direct Pick-Up Coping (PYRDC, LOT 2306337); 4.5 mm Regular Direct Pick-Up Coping (PGRDC, LOT 2404528); analogs: PYIA and PGIA	Cr–Ni alloy (Morelli); Pattern Resin LS (GC America); Putty Base/Catalyst (Ivoclar Vivadent); Type IV dental stone
G3	Conventional	Closed metal tray (Rimlock)	No	3.5 mm Snap Coping (PYRSC, LOT 2003054); 4.5 mm Snap Coping (PGRSC, LOT 2005286); analogs: PYIA and PGIA	Putty Base/Catalyst (Ivoclar Vivadent); Type IV dental stone
G4	Digital	—	No	3.5 mm Snap Scan Body, 11 mm (PYSSB11, LOT 2205552); 4.5 mm Snap Scan Body, 8 mm (PGSSB8, LOT 2401691); analogs: PYIA and PGIA	Intraoral scanner: Primescan (Dentsply Sirona)
G5	Digital	—	No	3.5 mm Titanium Scan Body (PYTSB, LOT 2404846); 4.5 mm Titanium Scan Body (PGTSB, LOT 2403992); analogs: PYIA and PGIA	Intraoral scanner: Primescan (Dentsply Sirona)

## Data Availability

All data are included in the manuscript.
